# Marker-Assisted Selection for Disease Resistance in Potato Breeding in the Ural Region of Russia (2018–2025): Comprehensive Genotyping and Validation of Key Resistance Markers

**DOI:** 10.3390/ijms27020855

**Published:** 2026-01-15

**Authors:** Georgiy A. Lihodeevskiy, Elena P. Shanina, Maria A. Stafeeva, Vadim F. Akhmetkhanov, Arina V. Shalaeva

**Affiliations:** Ural Federal Agrarian Research Center, Ural Branch of the Russian Academy of Science, 620142 Yekaterinburg, Russia; shanina08@yandex.ru (E.P.S.);

**Keywords:** *Solanum tuberosum*, *Phytophthora infestans*, *Globodera rostochiensis*, *Synchytrium endobioticum*, potato pathogens, SCAR markers, PCR genotyping, resistance breeding, molecular diagnostics

## Abstract

Marker-assisted selection (MAS) is a key tool in modern potato breeding for developing resistant varieties. This study aimed to evaluate the efficiency of molecular markers for selecting resistance to major pathogens in Ural (Russian Federation) potato breeding material. From 2018 to 2025, a total of 1212 hybrids and varieties were genotyped using 12 SCAR (sequence-characterized amplified regions) markers associated with resistance to potato wart (*Synchytrium endobioticum*), late blight (*Phytophthora infestans*), cyst nematodes (*Globodera* spp.), and viruses (PVX, PVY). The most frequent markers were TG689, N127, N195, and NL25. Phenotypic validation on more than 100 hybrids confirmed strong predictive power for NL25, TG689, and N195 markers in selecting resistance to wart disease and nematodes. In contrast, markers Rpi-blb1 and Rpi-sto1 for late blight did not show significant associations in this population. The results demonstrate the high diagnostic value of NL25, TG689, and N195 markers for MAS in Ural breeding programs, supporting their use for efficient selection of resistant genotypes.

## 1. Introduction

In modern breeding practice, a significant role is given to genetic technologies. They enable the identification of economically valuable traits in organisms, such as yield, accumulation of biologically active substances, and resistance to biotic and abiotic stresses. Among the methods widely used in Russia are the polymerase chain reaction (PCR) and gel electrophoresis. Their use ensures rapid and accurate detection of genetic determinants for target traits in agricultural crops, including potatoes, which determines their indispensability in the breeding process.

Marker-assisted selection (MAS) is one of the most widely used methods in modern agricultural practice. In potato breeding, this technology plays a key role by enabling the efficient selection of hybrids possessing target agronomic traits and enhanced resistance to pathogens. Genotyping methods allow for the identification of potato species, varieties, and hybrids carrying genetic markers associated with resistance to a wide range of infectious agents, including viral [1,2], bacterial [3], nematode [4,5], and fungal diseases [6,7]. The integration of marker-assisted selection into breeding programs contributes not only to increased yield and improved tuber quality but also to a significant reduction in the crop’s susceptibility to diseases [8,9,10].

Modern breeding practice utilizes a significant arsenal of molecular markers associated with potato resistance to phytopathogens. A substantial portion of the markers used for detecting resistance belongs to the SCAR (sequence-characterized amplified regions) category. A SCAR marker is a specific genomic DNA fragment, defined by its nucleotide sequence and localized to a precise locus, which is amplified in a polymerase chain reaction (PCR) using unique primers [11]. This class of markers is recognized as a highly effective tool in plant breeding, as it provides researchers with the ability to reliably and rapidly identify alleles of genes that determine resistance to diseases and pest damage. The use of SCAR markers underlies the accelerated development of variety identification with complex pathogen resistance, which is a key factor in increasing yield and improving product quality indicators.

The causal agent of potato wart disease, the obligate biotrophic soil fungus *Synchytrium endobioticum* (phylum *Chytridiomycota*), is among the most dangerous pathogens, capable of causing significant yield losses reaching 50–100% [12]. The pathogenesis is characterized by the formation of galls on tubers. The high virulence of *S. endobioticum* is due, in particular, to the exceptional resilience of its winter spores (zoosporangia), which remain viable in soil for over 40 years [13], as well as the absence of effective chemical control measures. The widespread prevalence of the pathogen in potato-growing regions, including the Russian Federation, makes breeding resistant varieties a critically important task. To enable efficient selection of resistant genotypes, the use of molecular markers linked to resistance genes plays a key role. One of the most significant resistance genes is *Sen1*. Using the marker NL25, a specific allele of the *Sen1* gene associated with resistance to the pathogen can be identified [6,14]. The efficacy of this marker and the corresponding allele in resistance breeding has been repeatedly validated in independent studies [15,16,17].

Late blight, caused by the oomycete *Phytophthora infestans*, remains the most economically significant disease of potato, leading to substantial yield losses globally [18,19,20,21,22]. The primary strategy for late blight control continues to be multiple fungicide applications, with the number reaching 10–15 in Northern Europe and up to 25 times per growing season under wet conditions [19]. However, intensive chemical use promotes the selection of more aggressive pathogen strains [23,24]. Consequently, a key direction for reducing pesticide load is the development of varieties with durable resistance. Wild Mexican potato species serve as a promising source of such traits. In particular, the broad-spectrum gene *Rpi-blb1* was identified in the diploid species *S. bulbocastanum* [25,26]. Due to interspecific incompatibility, its introgression into cultivated potato (*S. tuberosum*) was achieved using somatic hybridization methods [27]. A functional homolog of this gene, *Rpi-sto1*, was discovered in the allotetraploid species *S. stoloniferum* [28]. Both genes (*Rpi-blb1* and *Rpi-sto1*) have been successfully mapped [25,29], and specific PCR markers have been developed for their detection in breeding material [28,30]. According to current knowledge, pyramiding several resistance genes in a single variety is recommended to enhance the effectiveness and durability of resistance [31].

Cyst-forming nematodes of the genus *Globodera* (*G. rostochiensis* and *G. pallida*) are among the most dangerous pests of potato, causing significant yield reductions worldwide [32]. Yield losses, resulting from nematode parasitism on the root system and tubers, leading to a decrease in their size, can reach 20–70% [33]. Cultivation of resistant varieties is the most environmentally safe and effective method of control. Breeding such varieties is based on the use of key resistance genes. The dominant allele of the *Gro1* gene, introgressed from *Solanum spegazzinii* and mapped to chromosome VII, confers resistance to *G. rostochiensis* [34]. Specifically, the *Gro1-4* allele confers resistance to pathotypes Ro1-Ro5 of this species [35,36]. The *H1* gene, sourced from *S. tuberosum* subsp. *andigena* mediates a hypersensitive response to pathotypes Ro1 and Ro4 of *G. rostochiensis* [37]. In turn, the *Gpa2* gene determines resistance to some populations of *G. pallida* [38,39,40]. Specific molecular markers are used to identify these genes in breeding programs: Gro1-4-1 for the *Gro1* gene [4]; TG689 [41] and N195 [4] for the *H1* gene; and Gpa2-2 for the *Gpa2* gene [4]. Notably, markers TG689 and N195 detect the same *H1* gene but appear to be different variants for their tagging, which can lead to segregation in analyses [42,43,44]. Thus, pyramiding alleles of the *Gro1*, *H1*, and *Gpa2* genes in modern cultivars is a strategically important direction for creating an effective and durable resistance system to cyst-forming nematodes.

In addition to fungal and nematode diseases, viral infections pose a substantial threat to potatoes. Potato virus X (PVX) is one of the most widespread pathogens, causing global economic damage with estimated yield losses of up to 20% [45]. The PVX symptom complex includes mosaic, chlorosis, leaf rugosity, and necrosis, leading to significant suppression of photosynthetic activity and, consequently, reduced plant productivity. The infection negatively affects tuberization, resulting in the formation of small and underdeveloped tubers. The latent (asymptomatic) form of infection poses a particular epidemic threat, where the virus persists in the host plant without visible symptoms [46]. This property of PVX significantly complicates visual diagnosis and promotes the uncontrolled spread of the pathogen through infected seed material. Resistance to PVX is conferred by the *Rx* gene. This gene was identified in *Solanum tuberosum* subsp. *andigena*, annotated on chromosome XII near *Gpa2* [47], and can be detected using the RxSP marker [2].

Potato virus Y (PVY) is recognized as one of the most economically significant pathogens of the crop, capable of causing yield losses of up to 70%. The infection leads to tuber development disorders, exacerbating economic damage [48]. Therefore, developing varieties with genetic resistance to PVY is a priority in breeding [49]. A high level of resistance to PVY is provided by the *Ry_adg_* gene (source—*S. tuberosum* subsp. *andigena*), *Ry_sto_* (*S. stoloniferum*), and *Ry_chc_* (*S. chacoense*), which confer extreme resistance, i.e., the ability to suppress the replication of a wide range of virus strains [37,50,51,52]. These genes have been mapped to chromosomes XI, XII, and IX, respectively [53,54,55]. Based on these studies, breeding markers have been developed: RYSC3 for the *Ry_adg_* gene [56], YES3-3A for *Ry_sto_* [57], and Ry186 for *Ry_chc_* [58]. Mixed viral infections pose a particular danger. Co-infection with PVX and PVY, as well as PVX and Potato leafroll virus (PLRV), leads to a synergistic effect and the most significant yield losses [45,59,60]. Consequently, a strategic objective of modern breeding is to pyramid resistance genes to several pathogens in a single cultivar [61,62]. Markers NL25, Gpa2-2, Gro1-4-1, N195, YES3-3A, RYSC3, Rx1, Rpi-blb1, and Rpi-sto1 are included in the Russian genetic profile of potato varieties [63].

## 2. Results

### 2.1. Genotyping of Hybrids and Varieties of Ural Breeding

**Figure 1:**
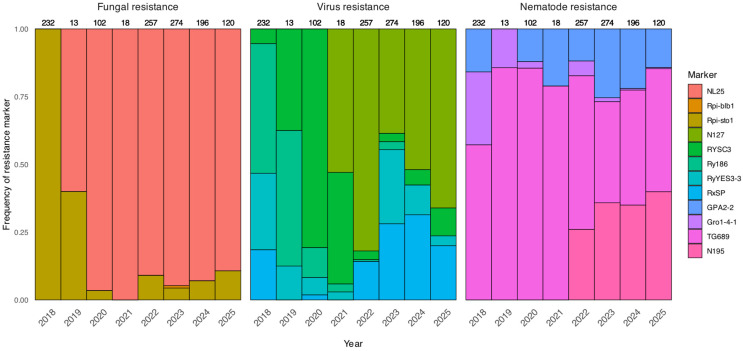
Frequency of resistance markers in potato samples across years (2018–2025). The bar charts show the percentage of samples positive for each molecular marker divided by group associated with resistance to fungal, viral and nematode pathogens. The total number of samples analyzed each year is indicated above the corresponding group of bars.

Between 2018 and 2025, a total of 1212 hybrids and varieties from the collection of the Potato Breeding and Seed Production Center were genotyped. In the initial years, the research focused on screening the plant material collection for donor genotypes (Table S1). We have only recently begun implementing marker-assisted selection (MAS) as a methodology for selecting hybrids for further breeding work [64,65,66]. Genotyping began in 2018 with 9 markers: Rpi-blb1, Rpi-sto1, RYSC3, Ry186, RyYES3-3, RxSP, Gpa-2-2, Gro1-4-1, TG689. The NL25 marker was added in 2019, followed by the N127 marker in 2021 (Figure 1). Starting from 2022, genotyping has been conducted for 12 resistance markers (with the N195 marker introduced into the workflow) on at least 100 samples per year. The number of hybrids and varieties with complete genotype data is 629 (Table 1). The most frequently encountered marker in the studied samples was TG689, detected in 84.5% of the plants. Resistance to potato wart disease, a trait under intensive selection at the Center, was identified in 70.6% of the plants. The markers N127 and N195 were also quite prevalent, found in 83.4% and 70.9% of plants, respectively. The rarest marker was Rpi-blb1, observed in only a single sample.

**Figure 2:**
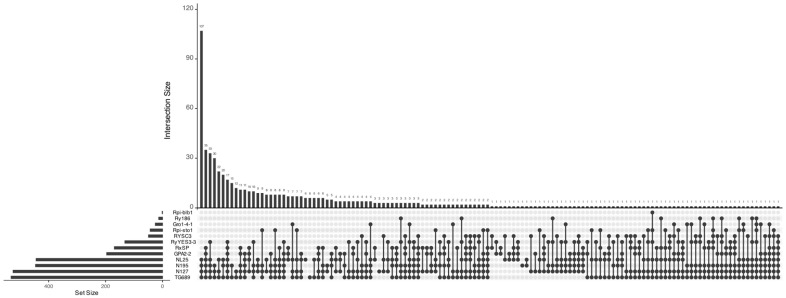
UpSet diagram displaying the distribution and intersections of molecular markers for resistance genes in the studied potato plant collection. The horizontal axis shows the size of individual sets (number of plants carrying each marker), and the vertical axis shows the size of intersections (number of plants with specific marker combinations).

At this stage of the project, gene pyramiding is not the primary objective. Nevertheless, we have identified 107 plants whose genotypes simultaneously contain the markers TG689, N127, N195, and NL25 (Figure 2). Overall, these four markers are the most prevalent in the studied population. Rare markers (Rpi-blb1, Ry186, Gro1-4-1, Rpi-sto1) are present in only a small number of samples, yet some of them form complex genotype combinations with other markers. The most notable multiple resistances involve the markers RyYES3-3, RxSP, and Gpa-2-2 (found in 10 to 35 plants depending on the specific intersection). In total, there are 5 samples containing any 8 markers simultaneously, 26 samples with 7 markers, and 72 samples with 6 markers.

The evaluation of associations between primer pairs revealed several significant linkages (Table 2). Strong associations (Cramér’s V > 0.3, *p* < 0.01) were established for the pairs TG689–N195 and Gpa-2-2–RxSP, and moderate associations (Cramér’s V > 0.15) for Ry186–RyYES3-3 and RYSC3–RxSP. All these pairs exhibit positive associations between the markers, based on odds ratios (OR > 1).

### 2.2. Validation of Genotyping Results

Testing for resistance to *S. endobioticum* (D1) and *G. rostochiensis* (Ro1) was conducted as part of the state variety trials. Over 9 years, 242 hybrids underwent this testing.

Resistance phenotypes were distributed as follows: for potato wart disease, 211 resistant, 15 moderately resistant, 16 susceptible; for the golden nematode, 183 resistant, 13 moderately resistant, 45 susceptible.

**Figure 3:**
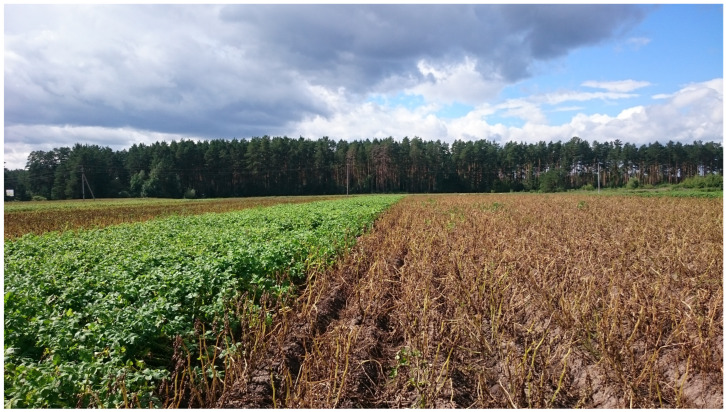
An example of high resistance of a new potato cultivar to late blight under *Phytophthora infestans* epiphytotic conditions. On the right, complete plant death of susceptible potato cultivars is shown. The plant development phase corresponds to stages 3 and 4 according to Kacheyo et al. 2021 [67].

During late blight epiphytotics in the experimental fields of the Ural Federal Agricultural Research Center of the Ural Branch of the Russian Academy of Sciences (see Figure 3 for an example of the field conditions), field observations identified 14 potato hybrids/varieties as resistant and 6 hybrids as susceptible to *P. infestans*.

Among the six tested molecular markers, three demonstrated a statistically significant (*p* < 0.001) and strong positive association with the corresponding resistance phenotype (Table 3). The most effective was the NL25 marker: its presence predicted resistance with a positive predictive value (PPV) of 98.5%, and an odds ratio (OR) of 48.75 (95% CI: 10.2–233.8). The TG689 marker demonstrated the highest sensitivity (0.93), although its specificity was moderate (0.60). The N195 marker also showed a high PPV (93.3%) with moderate sensitivity and specificity. The markers Gro1-4-1, Rpi-sto1, and Rpi-blb1 did not show a statistically significant association with resistance (*p* > 0.05) and possessed low diagnostic characteristics, which preclude their practical use for selection within this population.

## 3. Discussion

The present study reports the results of multi-year (2018–2025) genotyping of a large potato collection using marker-assisted selection to identify resistance to the most important pathogens affecting the Ural region. Over the course of the project, more than 1000 potato hybrids and varieties were genotyped, with complete data available for all 12 markers in 629 plants. This study presents the first comprehensive report integrating multi-year genotyping data with phenotypic validation from the Ural potato breeding program. While preliminary methodological approaches and screening results for subsets of the material were reported earlier [64,65,66], the consolidated dataset, full-scale marker-trait association analysis, and validation metrics presented here are original and have not been published previously.

The most common markers, each present in more than 400 plants, are TG689, N127, N195, and NL25. According to Russian sources, these are among the most prevalent markers [15]. In 107 plants, or about one-sixth of all plants with complete genotypic data, these same markers occur simultaneously. Overall, within the studied plant group, there are over a hundred possessing 5 or more resistance markers, thus creating favorable conditions for further breeding work.

The identified significant association between TG689 and N195 is not surprising, as both markers are associated with the H1 gene. High association degrees are also characteristic of the Gpa-2-2–RxSP pair, which forms a linkage group. For the Ry186–RyYES3-3 pair, a high and significant OR was found, but with a wide CI due to the rarity of combinations involving the Ry186 marker. The discovered associations between TG689–N127, N195–N127, Gpa-2-2–RYSC3, RYSC3–RxSP, and RyYES3-3–N127 are of interest; these are associations of moderate strength, even though the markers belong to different chromosomes (Table 4). This result may indicate a systemic breeding structure within the collection.

Our results demonstrate the high efficacy of the NL25, TG689, and N195 markers for predicting field resistance to major potato pathogens. The NL25 marker, associated with the *Sen1* gene conferring resistance to potato wart disease, showed exceptionally high positive predictive value (PPV = 98.54%) and an odds ratio (OR = 48.75), indicating its particular diagnostic value in our population. These findings are consistent with GWAS analysis data from Polish and German cultivars, where the NL25 marker was also identified as one of the most reliable for detecting resistance to pathotype D1 [70].

The TG689 and N195 markers, both designed to detect the *H1* gene for resistance to the golden potato cyst nematode, demonstrated high sensitivity (Se > 0.8) and moderate specificity (Sp ≥ 0.60), along with high predictive ability. Similar observations were made in an earlier breeding study in the Czech Republic, where the TG689 marker showed over 90% correspondence with the phenotypic expression, which aligns with our PPV of 91.04% [10]. Experience from Russian collections (Institute of Plant Industry collection, VIR), where 113 domestic and related potato cultivars were analyzed, showed that the NL25 marker provides high accuracy for detecting resistance to *S. endobioticum*. However, the predictive ability of markers for the *H1* and *Gro1-4* genes was significantly more variable, requiring the use of several markers in combination for reliable identification of the resistant genotype [15]. In our study, the Gro1-4-1marker proved uninformative for *G. rostochiensis* (pathotype Ro1), most likely due to its relatively low frequency in our material.

An unexpected result was obtained during the validation of markers for resistance to late blight (*Phytophthora infestans*). The Rpi-blb1 and Rpi-sto1 markers, despite their widespread recognition and successful use in European breeding programs, did not demonstrate a statistically significant association with field resistance in our population. This result can primarily be explained by the small sample size of only 20 hybrids/varieties. This observation does not indicate the ineffectiveness of the markers themselves but rather points to their rarity in the Ural breeding material and possibly to different sources of resistance to *P. infestans* in the local gene pool. In future research, it would be useful to increase the number of markers associated with late blight resistance, in particular to include Rpi-R8, as the observed resistance may be conditioned by other loci [31,71].

## 4. Materials and Methods

The study utilized hybrid material from the breeding nurseries of the Potato Breeding and Seed Production Center at the Ural Federal Agricultural Research Center of the Ural Branch of the Russian Academy of Sciences.

For DNA extraction from plant samples, the reagent kit “Sorb-GMO-B” (“Syntol,” Moscow, Russia) was employed. PCR was performed using the primers listed in Table 5, with conditions corresponding to the original sources for each marker. A Taq DNA polymerase kit (“Eurogen,” Moscow, Russia) was used for the PCR. The GBSS-3 and BCH-2 markers served as positive controls for DNA extraction. The PCR products were visualized via gel electrophoresis.

Resistance testing of potato hybrids and varieties against *S. endobioticum* pathotype 1(D1) and *G. rostochiensis* (pathotype Ro1) was conducted at the All-Russian Research Institute of Starch and Starch-Containing Raw Materials Processing—a Branch of the Russian Potato Research Centre. Based on field observations during epiphytotics, potato hybrids showing resistance and susceptibility to *P. infestans* were identified.

Genotyping results were entered into Microsoft Excel spreadsheets and analyzed using R v4.5.2 [73]. Results for all markers were coded as 1 for presence and 0 for absence in the genotype. The strength of association between marker pairs was assessed using Cramér’s V, implemented via the assocstats() function from the vcd package [74], while the direction of association was evaluated using the oddsratio() function from the epitools package [75].

Phenotypes were defined as follows: for *S. endobioticum* and *P. infestans*: Resistant and Susceptible; for *G. rostochiensis*: Resistant, Moderately resistant, and Susceptible. For binary categorical analysis, the Resistant and Moderately resistant categories were combined into a single group.

To assess the genotype-phenotype association, contingency table analysis with Odds Ratio (OR) calculation was performed using the epi.2by2() function from the epiR package v2.0.89 [76]. Agreement between genotype and phenotype was evaluated using Weighted Kappa via kappa2(weight = “squared”) from the irr package v0.84.1 [77], and the significance of associations was tested with the chi-square statistic using the chisq.test() function.

Visualization was performed in R using the ggplot2 v4.0.1 [78] and UpSetR v1.4.0 [79] packages.

## Figures and Tables

**Table 1:** Total number of resistant plant forms by molecular marker identified over the entire period.

NL25	Rpi-blb1	Rpi-sto1	N127	RYSC3	Ry186	RyYES3-3	RxSP	Gpa-2-2	Gro1-4-1	TG689	N195
Genotyping results from 2018 to 2025											
628	1	133	649	162	75	177	278	341	114	926	493
With full genotypes											
444	1	43	525	49	13	132	169	196	25	532	446

**Table 2:** Assessment of pairwise associations of molecular markers: Odds Ratios, Cramér’s V coefficient and *p*-values for identifying significant linkages between loci.

Marker Pairs	OR (95% CI)	Cramér’s V	*p*-Value
TG689–N195	8.5 (5.1:14.3)	0.39	<0.001
TG689–N127	2.3 (1.3:3.9)	0.13	0.002
N195–N127	1.9 (1.2:3.1)	0.12	0.004
Gpa-2-2–RYSC3	2.5 (1.3:4.7)	0.12	0.003
Gpa-2-2–RxSP	16.2 (10.4:25.7)	0.57	<0.001
RYSC3–RxSP	3.5 (1.8:6.5)	0.17	<0.001
Ry186–RyYES3-3	22.4 (4.8:209.5)	0.23	<0.001
RyYES3–3-N127	2.8 (1.4:6.3)	0.12	0.003

**Table 3:** Association of molecular markers with phenotypic resistance.

Marker	N	Marker+/Resistance (PPV, %)	OR (95% CI)	*p*-Value	Sensitivity	Specificity	Kappa
NL25	168	135/137 (98.5)	48.75 (10.2:233.8)	<0.001	0.88	0.87	0.51
TG689	241	183/201 (91.0)	21.12 (9.3:47.9)	<0.001	0.93	0.6	0.56
N195	120	83/89 (93.3)	9.99 (3.4:29.8)	<0.001	0.82	0.68	0.40
Gro1-4-1	241	24/31 (77.4)	0.76 (0.3:1.9)	0.74	0.12	0.84	−0.01
Rpi-sto1	20	12/18 (66.7)	n/c	0.33	0.86	0	−0.18
Rpi-blb1	20	1/1 (100.0)	n/c	0.50	0.07	1	0.04

n/c—not calculable due to a zero cell in the contingency table.

**Table 4:** Chromosomal organization of potato resistance genes to pathogens.

Marker	Gene/Resistance	Chromosome	Source
TG689 N195	H1 (*G. rostochiensis*)	V	[68]
N127	*PLRV.1* (PLRV)	XI	[69]
RYSC3	*Ry_adg_* (PVY)	XI	[56]
Ry186	*Ry_chc_* (PVY)	XI	[56]
RyYES3-3	*Ry_sto_* (PVY)	XI	[14]
Gpa-2-2	*Gpa2* (*G. pallida*)	XII	[47]
RxSP	*Rx1* (PVX)	XII	[47]

**Table 5:** List of primers, markers, and their target genes.

Gene	Trait	Marker _Product size_	Primer Pairs (5′–3′)	Source
*Sen1*	Resistance to potato wart disease	NL25_1400_	F: TATTGTTAATCGTTACTCCCTC R: AGAGTCGTTTTACCGACTCC	[14]
*Rpi-blbl*	Resistance to late blight.	Rpi-blb1_821_	F: AACCTGTATGGCAGTGGCATG R: GTCAGAAAAGGGCACTCGTG	[28]
*Rpi-sto1*		Rpi-sto1_890_	F: ACCAAGGCCACAAGATTCTC R: CCTGCGGTTCGGTTAATACA	[30]
*PLRV1*	Resistance to leaf roll virus	Nl27_1164_	F: TAGAGAGCATTAAGAAGCTGC R: TTTTGCCTACTCCCGGCATG	[69]
*Ry_adg_*	Resistance to PVY	RYSC3_321_	F: ATACACTCATCTAAATTTGATGG R: AGGATATACGGCATCATTTTTCCGA	[56]
*Ry_chc_*		Ry186_587_	F: TGGTAGGGATATTTTCCTTAGA R: GCAAATCCTAGGTTATCAACTCA	[1]
*Ry_sto_*		YES3-3A_341_	F: TAACTCAAGCGGAATAACCC R: AATTCACCTGTTTACATGCTTCTTGTG	[5]
*Rx1*	Resistance to PVX	RxSP_1230_	F: ATCTTGGTTTGAATACATGG R: CACAATATTGGAAGGATTCA	[2]
*Gpa2-2*	Resistance to *G. pallida*	Gpa2-2_452_	F: GCACTTAGAGACTCATTCCA R: ACAGATTGTTGGCAGCGAAA	[4]
*Gro1-4*	Resistance to *G. rostochiensis*	Gro1-4-1_602_	F: AAGCCACAACTCTACTGGAG R: GATATAGTACGTAATCATGCC	[4]
*H1*		TG689_141_	F: TAAAACTCTTGGTTATAGCCTAT R: CAATAGAATGTGTTGTTTCACCAA	[41]
		N195_337_	F: TGGAAATGGCACCCACTA R: CATCATGGTTTCACTTGTCAC	[4]
*GBSS*	Granule-bound starch synthase	GBSS-3_853_	F: AAAGGAGGCTCTTCAAGCAG R: TGCAAGAGCTCTAGCAACTG	[4]
*BCH*	Beta-carotene hydroxylase	BCH-2_290_	F: CATGACATAGTTTGAATTTTGAGTC R: GCTTTGGCGCTGCCGTAAGTT	[72]

## Data Availability

The original contributions presented in this study are included in the article/Supplementary Materials. Further inquiries can be directed to the corresponding author.

## Supplementary Materials

The following supporting information can be downloaded at: https://www.mdpi.com/article/10.3390/ijms27020855/s1.

## Abbreviations

The following abbreviations are used in this manuscript:

MAS	Marker-Assisted Selection
SCAR	Sequence-Characterized Amplified Region
PVX	Potato Virus X
PVY	Potato Virus Y
PLRV	Potato Leafroll Virus
OR	Odds Ratio
CI	Confidence Interval
PPV	Positive Predictive Value
Se	Sensitivity
Sp	Specificity
